# In Situ Synthesis of Ternary Ni-Fe-Mo Nanosheet Arrays for OER in Water Electrolysis

**DOI:** 10.3390/molecules30010177

**Published:** 2025-01-04

**Authors:** Zhi Lu, Yifan Guo, Shilin Li, Jiaqi Ding, Yingzi Ren, Kun Tang, Jiefeng Wang, Chengxin Li, Zishuo Shi, Ziqi Sun, Hongbo Meng, Guangxin Wang

**Affiliations:** 1 School of Materials Science and Engineering, Henan University of Science and Technology, Luoyang 471003, China; guoyifan20010310@163.com (Y.G.); lishilin0402@163.com (S.L.); d2827868483@163.com (J.D.); m18147385505@163.com (Y.R.); tang01101344@163.com (K.T.); 18739029558@163.com (C.L.); 18338072696@163.com (Z.S.); 18862989942@163.com (Z.S.); 2 Henan Engineering Research Center for High Purity Materials and Sputtering Targets, Luoyang 471003, China; 3 School of Mechanical Engineering, Anyang Institute of Technology, Anyang 455099, China; wangyufan2007@163.com; 4 Luoyang Crystal Union Photoelectric Materials Co., Ltd., Luoyang 471000, China; hongbomeng@163.com

**Keywords:** nanosheet arrays, Ni foam, OER, water electrolysis

## Abstract

Water electrolysis is a promising path to the industrialization development of hydrogen energy. The exploitation of high-efficiency and inexpensive catalysts become important to the mass use of water decomposition. Ni-based nanomaterials have exhibited great potential for the catalysis of water splitting, which have attracted the attention of researchers around the world. Here, we prepared a novel Mo-doped NiFe-based layered double hydroxide (LDH) with a nanoarray microstructure on Ni foam. The doping amount of Mo can significantly change the microstructure of the electrocatalysis, which will further affect the oxygen evolution reaction (OER) performance of water splitting. This novel nanomaterial required only an overpotential of 227 mV for 10 mA cm^−2^ and a Tafel slope of 54.8 mV/dec in 1 M KOH. Meanwhile, there was no Mo, and the NiFe-LDH needed 233 mV to attain to 10 mA cm^−2^. Compared to the NiFe-LDH without Mo, the NiFeMo-LDH nanosheet arrays exhibited enhanced activities with 17.1 mV/dec less Tafel in OER. The good performance of the electrocatalyst is ascribed to the special nanosheet arrays and the heterostructure of the Ni-Fe-Mo system. These features help to increase the active surface, enhancing the efficient charge transfer and the reactive activity in OER.

## 1. Introduction

Gradual depletion of traditional fossil energy resources and the looming greenhouse effect have attracted the urgent demand of sustainable and environmentally friendly energy resource uses [1,2]. Ascribed to zero pollution, lightweight advantages, high-energy density, and ease of transport by pipeline or compressed container, hydrogen is thought of as a very promising substitution to replace traditional energy sources [3,4]. Because of its low proportion in the air, it is costly to separate H_2_ from the air, so most of the industrial H_2_ acquired is produced from reliance on traditional fossil energy. This will cause serious environmental pollution and enormous carbon emissions [5]. The hydrolysis of water, which is simple, environmentally friendly, and sustainable, is supposed as one of hopeful paths used in commercial processes for acquiring H_2_ [6]. Water decomposition can be understood as two half reactions, including an OER and a hydrogen evolution reaction (HER), whereas an OER is more crucial to water hydrolysis [7,8]. An OER was considered that contained four electrons transport and the production of some intermediates, which the restricted process of the overall decomposition process [9,10]. The energy of a catalytic reaction is always supplied by electric power, where the economic method is converted from solar and wind energy.

An OER is always sluggish and needs high overpotential to activate it [11,12]. To lower the overpotential and energy barrier of an OER, electrocatalyst is one of the optimal choices [13,14]. Nowadays, many kinds of electrocatalysts have been designed, which include among them some noble-metals-based electrocatalysts, such as Pt and Ru compounds that express high activity during HERs and OERs; however, their being costly as raw materials seriously hinders their industrialization [15]. Thus, the developing of a high cost-effective catalyst become an urgent demand. So far, considerable work has been put into designing transitional metal (Ni, Co, Fe, and Cu) hydroxides, which have showed remarkably effects when applied in OERs [16]. Transition metals—for instance, Ni-, Co-, and Fe-based layered double hydroxides (LDHs)—are cheap to synthesis, and their special microstructure can supply abundant reactive sites for an OER, which contributes to the catalytic performance [17,18]. Among them, Ni-based binary and ternary LDHs possess a heterogeneous microstructure, whose electronic structure is more active and shows excellent electrocatalytic activities [17].

Through doping other atoms, the OER performance of nickel base LDH was enhanced remarkably [19,20]. This can be ascribed to the regulation of the structure of the valence electron, which help to enhance the charge transport of the electrocatalyst [20]. Li et al. doped vanadium in an NiV-based LDH using hydrothermal approaches, and the result showed that the band gaps of the electronic structure of the compounds were shorter; this contributed to the increase in active sites, acceleration of charge transfer, and the improvement in catalytic efficiency. A competitive overpotential of 195 mV and a Tafel slope of 42 mV/dec were obtained [21]. Wei et al. synthesized Ir-doped nickel-based hydroxide by electrochemical deposition. It was found that the hollow sites of the facial center cubic of the compound were filled precisely by Ir atoms, which changed the valence state of the compound, and the material showed a competitive overpotential of 228 mV at 10 mAcm^−2^ [22]. Long et al. synthesized a Co-doped FeNi-based LDH by adjusting the content of Co, and the electron structure of the compound was regulated. The microstructure of the material was changed, and the active sites were increased significantly, which enhanced the OER performance [23].

Optimization of the micromorphology is also conducive to the improvement of OER performance. Numerous electrocatalysts with special nanostructures such as nanosphere, nanowire and nanorod morphologies have been designed; they could supply plenty of open pores to catalytic reaction and displayed excellent OER performance outcomes [24,25]. Yu et al. constructed a NiMgAl-based LDH with the morphology of crossed nanosheets [26]. The special nanostructure could be adjusted by the ratio of the major elements and the content of dopant, which provided an excellent electron transport for the catalytic reaction and exhibited a high OER performance in application. Gao et al. designed an electrocatalyst with heterostructure of 2D and 1D compound structures: the nickel–vanadium composite had a 2D structure of nanoflakes, while the nickel–cobalt–phosphorus composite had a 1D structure of nanowires [27]. This heterostructure supplied abundant channels for ion exchange and mass transport, which significantly improved its performance on electrocatalytic decomposition of water.

Herein, a molybdenum-doped nickel–iron base LDH was prepared using the hydrothermal method, which is low-cost, helps to regulate the microstructure, and affects the performance of the electrocatalyst. The microstructure could be significantly regulated by adjusting the content of dopants. We adjusted the ratio of Ni/Fe/Mo and obtained different three-dimensional nanoarray morphologies that led to different performance outcomes. The as-prepared NiFeMo-based LDH exhibited a promising OER performance: it needed a competitive overpotential of 227 mV for 10 mA cm^−2^ and a Tafel slope of 54.8 mV/dec. Meanwhile, the 24 h chronoamperometry test at 1.54 V (vs RHE) showed that the current density–time curve was steady. After 1000 CV cycles, the LSV of the NiFeMo-based LDH showed only a little change when compared to that before the cycle test. It had an outstanding stability. Its superior performance could be owed to the sufficient active area provided by the special micromorphology and the synergistic effects of the major elements in the compounds. The NiFeMo-based LDH reveals the potential application in hydrogen production.

## 2. Results and Discussion

### 2.1. Characterization

**Figure 1:**
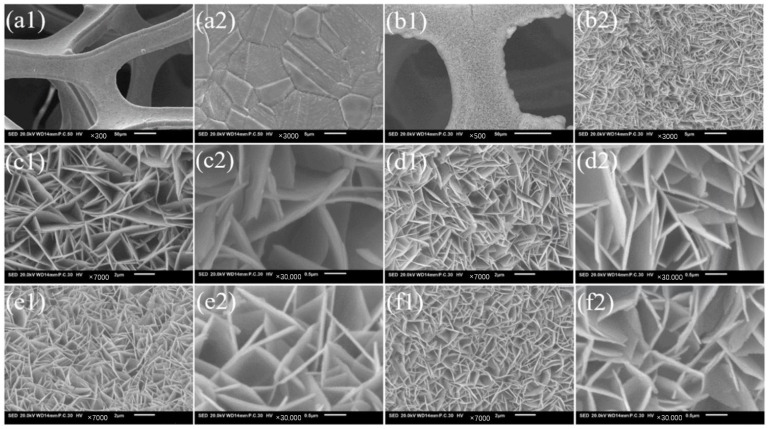
SEM of (**a1**,**a2**) pre-treated NF and (**b1**,**b2**) the surface after synthesis. Macro-scale images of (**c1**) NiFe-LDH/NF, (**d1**) NiFeMo_0.1_-LDH/NF, (**e1**) NiFeMo_0.2_-LDH, (**f1**) NiFeMo_0.3_-LDH nanosheet arrays. High magnification of (**c2**) NiFe-LDH/NF, (**d2**) NiFeMo_0.1_-LDH/NF, (**e2**) NiFeMo_0.2_-LDH, (**f2**) NiFeMo_0.3_-LDH.

Scanning electron microscope (SEM) images of the foam and NiFeMo-based LDH are shown in Figure 1. Before the synthesis, NF has a smooth surface, and the Ni crystal grains are clear (Figure 1(a1,a2)). However, after the synthesis, the surface of NF became rough, the prepared NiFeMo-based LDH electrocatalysts show a morphology of nanosheets (Figure 1(b1,b2)). There are obvious differences between the different composition of the specimens. From the low magnification images (Figure 1(c1,d1,e1,f1)) of the specimens, the density of the nanosheets can be seen to improve with increasing Mo concentrations. As seen in the high-magnification morphologies (Figure 1(c2,d2,e2,f2)), when there is no Mo, the nanosheets present thick and high porosity. When increasing the Mo concentrations, the nanosheets become thinner and their size reduces, being distributed more densely. This may affect the electrochemical performance of NiFeMo-based LDHs.

As shown in Table 1, “Length” means the average length of the nanosheets in Figure 1; “Thickness” means the average thickness of the nanosheets in Figure 1; “L/T” means the ratio of “Length” to “Thickness”. As can be seen, the average length of the NiFe-LDH, NiFeMo_0.1_-LDH, NiFeMo_0.2_-LDH, and NiFeMo_0.3_-LDH are 2.37 µm, 1.91 µm, 1.53 µm, and 1.22 µm. The average thickness of the NiFe-LDH, NiFeMo_0.1_-LDH, NiFeMo_0.2_-LDH, and NiFeMo_0.3_-LDH are 0.2 µm, 0.13 µm, 0.11 µm, and 0.09 µm, respectively. The L/T of NiFe-LDH, NiFeMo_0.1_-LDH, NiFeMo_0.2_-LDH, and NiFeMo_0.3_-LDH are 11.8, 14.7, 13.9, and 13.5. The parameters of the LDHs may be depend on the ratio of the cations, which thereby influence the property of the LDHs [28]. According to the analysis above, it could be found that with increasing Mo concentrations, the ratio of L/T presents earlier increases and later decreases. When the Ni: Fe: Mo ratios was 1:1:0.1, the ratio of L/T was the highest, which helped to improve the quantity of active sites and the electrochemical performance.

**Figure 2:**
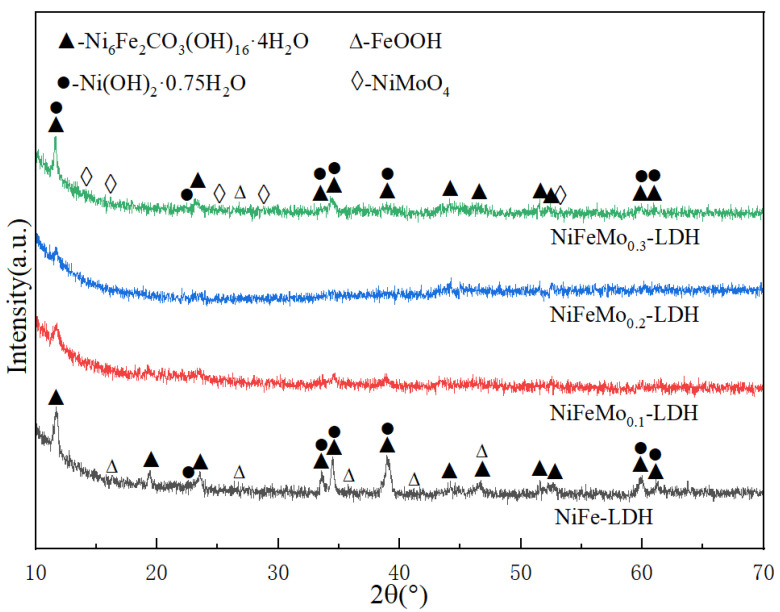
XRD patterns of NiFeMo-based LDH. The patterns of NiFe-LDH (black), NiFeMo_0.1_-LDH (red), NiFeMo_0.2_-LDH (blue) and NiFeMo_0.3_-LDH (green).

Figure 2 shows X-ray diffraction (XRD) patterns of the NiFeMo-based LDHs. The sharp diffraction peaks at 2θ = 11.63, 23.39, 34.47, and 59.98 correspond to (006), (0012), (204), and (220) of Ni_6_Fe_2_CO_3_(OH)_16_·4H_2_O (JCPDF No.26-1286). The peaks at 2θ = 11.35, 22.74, 33.46, 34.41, 38.77, and 59.98 correspond to (003), (006), (101), (012), (015), and (110) of Ni(OH)_2_·0.75H_2_O (JCPDF No.38-0715) [19,21]. Other peaks, such as 2θ = 33.41 and 35.90 correspond to the (400) and (211) of FeOOH (JCPDF No.18-0639), and 2θ = 14.30 and 28.82 correspond to the (110) and (220) of NiMoO_4_ (JCPDF No.33-0948). These prove that the NiFeMo-based LDH nanosheet arrays were successfully synthesized on the nickel foam [24,27].

**Figure 3:**
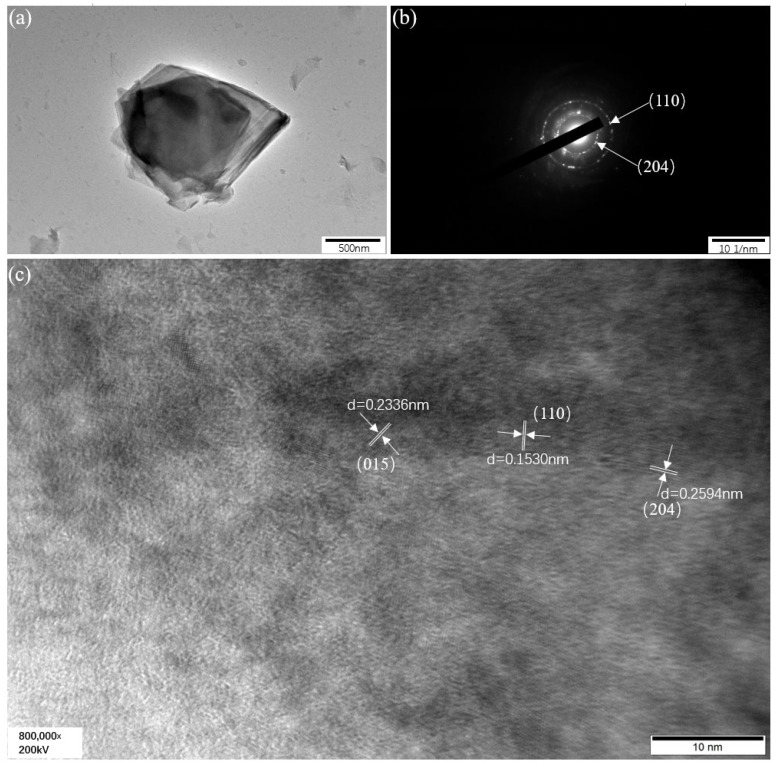
(**a**) TEM morphology, (**b**) SAED, (**c**) high-resolution image of NiFeMo_0.1_-LDH.

Transmission electron microscopy (TEM) would be conducive to deeply study the microstructures of materials. Figure 3 is the TEM analysis of NiFeMo_0.1_-based LDH. Figure 3a illustrates the ultrathin nanosheet of NiFeMo_0.1_-based LDH, which has a considerable surface area about 1.5 × 1 μm. This contributes to providing abundant active sites to electrocatalytic reactions. The pore volume and specific surface area of NiFeMo_0.1_-based LDH are shown in Table 2. As can be seen in the table, the specific surface area of NiFeMo_0.1_-based LDH is considerable, which can benefit the catalytic performance. The selected area electron diffraction (SAED) of the NiFeMo_0.1_-based LDH is shown in Figure 3b; it reveals the phase features of Ni_6_Fe_2_CO_3_(OH)_16_·4H_2_O (JCPDF No.26-1286) and Ni(OH)_2_·0.75H_2_O(JCPDF No.38-0715). The two diffraction rings in Figure 3b are indexed as (110) planes of Ni(OH)_2_·0.75H_2_O and (204) plane of Ni_6_Fe_2_CO_3_(OH)_16_·4H_2_O from outside to inside. The high-resolution TEM image in Figure 3c illustrates that the interplanar distances of the lattice fringes are 0.2594 nm, 0.1530 nm and 0.2336 nm, which are indexed as the (204) plane of Ni_6_Fe_2_CO_3_(OH)_16_·4H_2_O and the (110) and (015) planes of Ni(OH)_2_·0.75H_2_O. The crystallinity of the material can be further improved by a following sintering, wherein the pore volume and specific surface area of the NiFeMo-based LDH will be affected, which will affect the quantity of active sites and even the electrochemical performance.

**Figure 4:**
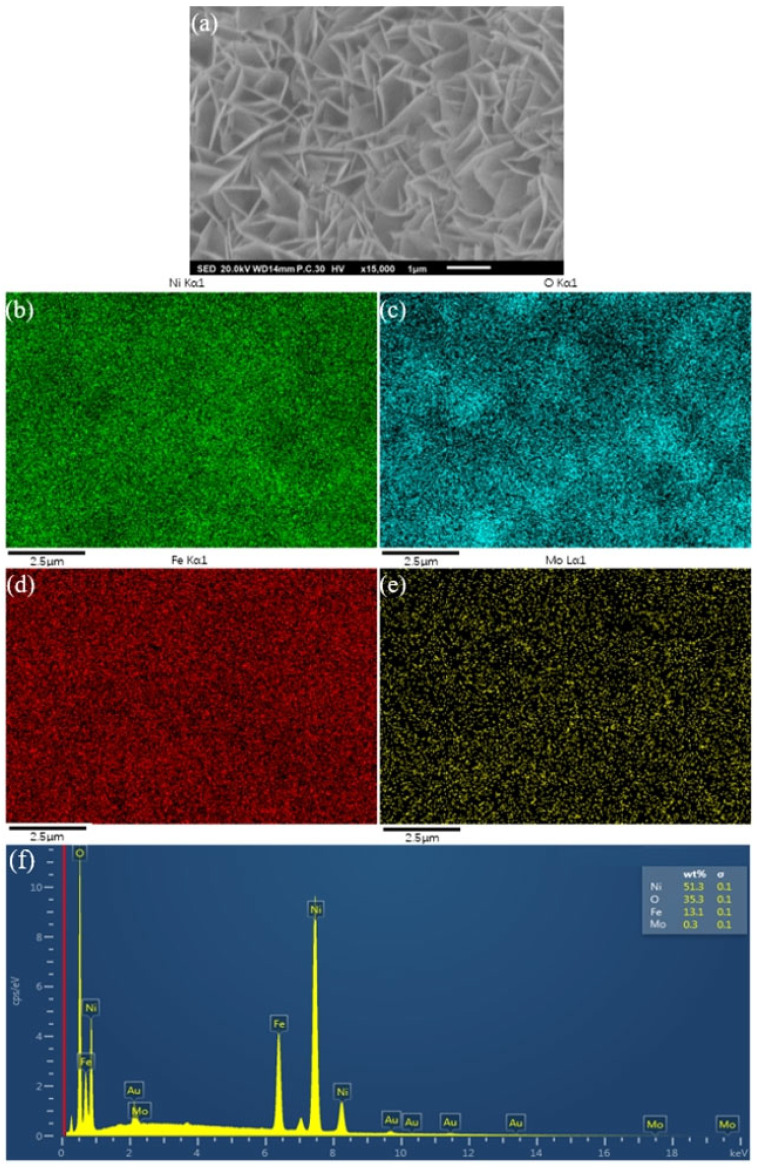
EDS of NiFeMo_0.1_-LDH nanosheet arrays. (**a**) SEM of NiFeMo_0.1_-LDH; the distribution of (**b**) Ni, (**c**) O, (**d**) Fe, (**e**) Mo, (**f**) EDS of NiFeMo_0.1_-LDH surface.

Energy dispersive spectrometer (EDS) analysis in Figure 4 shows the distribution of Ni, O, Fe, and Mo, which are uniform throughout the sample surface and thus can induce better stability in application.

### 2.2. X-Ray Photoelectron Spectroscopy (XPS) of NiFeMo-Based LDH

**Figure 5:**
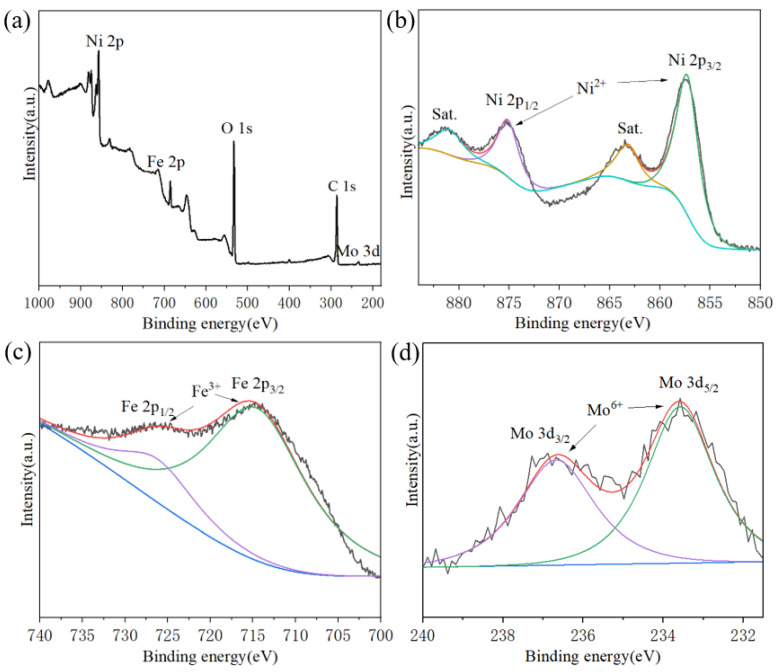
XPS of (**a**) survey spectrum, (**b**) Ni 2p, (**c**) Fe 2p, (**d**) Mo 3d of NiFeMo_0.1_-LDH. The red curves are the fitted curves of test data, the dark green and purple curves are the peaks of the main elements, the light green and yellow curves are the satellite peaks, the blue curves are the baseline of the fitted curves.

The XPS (Figure 5a) indicates the main elements in the samples as Ni, Fe, Mo, O, and C, and the atomic percentages analyzed are Ni 12.8%, Fe 3.3%, Mo 0.2%, O 31.9%, and C 44.8%. The spectra of Ni 2p, Mo 3d, Fe 2p, C 1s, and O 1s can be clearly identified.

The Ni 2p spectrum of NiFeMo_0.1_-LDH was separated to Ni 2p_1/2_ and Ni 2p_3/2_ (Figure 5b) at 875.5 eV and 857.4 eV along with satellite peaks at 863.3 eV and 881.3eV. This attributed to the valence state of Ni^2+^ [29]. The Fe 2p spectrum (Figure 5c) of NiFeMo_0.1_-LDH was separated to Fe 2p_1/2_ and Fe 2p_3/2_ at 726.4 eV and 713.7 eV, which attributed to the valence state of Fe^3+^. The Mo 3d spectrum was separated to Mo 3d_3/2_ and Mo 3d_5/2_ attributed to the valence state of Mo^6+^. According to the spectrum information, Ni: Fe: O (molar ratio) = 3.8:1:9.7 in NiFeMo_0.1_-LDH, which is similar to that in Ni_6_Fe_2_CO_3_(OH)_16_·4H_2_O and agrees with the results of the XRD and TEM above [30].

### 2.3. OER of NiFeMo-Based LDH

In this study, a three-electrode system was used to perform hydrolysis of water at 10mAcm^−2^, and the polarization curves were iR corrected.

**Figure 6:**
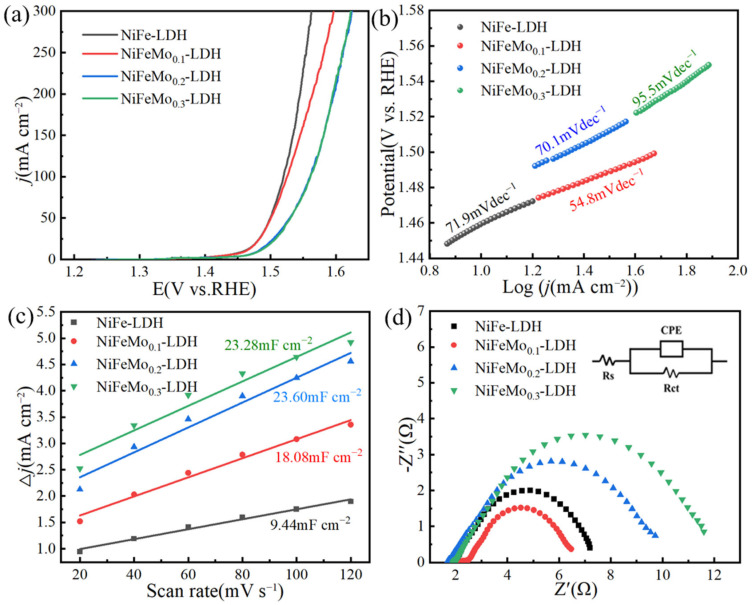
(**a**) LSV curves at 2 mVs^−1^. (**b**) Tafel slopes. (**c**) C_dl_ curves. (**d**) EIS with the equivalent circuit.

To assess the electrocatalytic activity of NiFeMo-based LDH, linear sweep voltammetry (LSV) evaluation was taken for OERs in electrolyte quantities of 1 M KOH over 2m V s^−1^ vs. reversible hydrogen electrode (RHE) (Figure 6a). The LSV plots shows that the overpotential of NiFeMo_0.1_-LDH (227 mV) was lower than the NiFe-LDH (233 mV), NiFeMo_0.2_-LDH (249 mV), and NiFeMo_0.3_-LDH (254 mV) at 10 mA cm^−2^. The lowest overpotential of the NiFeMo_0.1_-LDH can be attributed to the heterostructure interaction of Mo, Fe, and the matrix metal Ni in the electron structure of the NiFeMo_0.1_-LDH [31].

Figure 6b shows that the NiFeMo_0.1_-LDH possessed the lowest Tafel slope (54.8 mV/dec), possibly because of the firm electron coupling among the Ni, Fe, and Mo [32]. In contrast, the

Tafel slopes of the NiFe-LDH, NiFeMo_0.2_-LDH and NiFeMo_0.3_-LDH were measured as 71.9, 70.1, and 95.5 mV/dec. The lowest Tafel slope of the NiFeMo_0.1_-LDH indicates the faster charge transfer kinetics of the NiFeMo_0.1_-LDH in its OER. This can be attributed to its low overpotential and Tafel slope, meaning that the NiFeMo_0.1_-LDH can be a promising electrocatalyst with superior OER performance.

The electrochemical active surface area (ECSA) is used to assess the amounts of active sites during an OER, and it is important to electrocatalysis. The ECSA was obtained according to the bellow formula: ECSA = C_dl_/C_s_, where C_dl_ is the double layer capacitance of the electrocatalyst; C_s_ is the specific capacitance of the material to the nickel base material; and C_s_ is always 4 × 10^−2^mF · cm^−2^ [32]. The C_dl_ can be calculated from the results of a cyclic voltammetry (CV) test. The C_dl_ of the NiFeMo-based LDHs (Figure 6c) were significantly higher than the NiFe-LDH. This indicates the higher density of the catalytic active sites in the NiFeMo-based LDHs, which can be ascribed to the synergistic interaction of the Ni, Fe, and Mo [20].

As shown in the electrochemical impedance spectroscopy (EIS) results (Figure 6d), the NiFeMo_0.1_-LDH exhibited a smaller semicircle than the others, suggesting its higher conductivity, which greatly affects the electrocatalytic activity. The equivalent circuit consists of solution resistance (Rs), charge transfer resistance (Rct), and the constant phase element (CPE). The Rct value of the NiFeMo_0.1_-LDH (4.56 Ω) was lower than that of the NiFe-LDH (5.81 Ω), NiFeMo_0.2_-LDH (8.75 Ω), and NiFeMo_0.3_-LDH (10.53 Ω). The lowest impedance of the NiFeMo_0.1_-LDH indicates that the synergistic effect of the Ni, Fe, and Mo effectively improved the charge transport kinetics of the electrocatalyst [23].

During water electrolysis, the lattice oxygen atoms in the surface of the catalysts can be replaced by the oxygen atoms from the electrolyte. The lattice oxygen atoms are released from the bonding of M-O in the surface of the catalysts (M represent the main metal ions), and the oxidation states of the metal ions are consequently lowered [33,34]. At the beginning of an OER, OH^−^ in the solution is adsorbed to the surface of catalyst, which produces electrons and intermediate OH molecules. After that, the intermediate OH combines with the OH^−^ that came from the electrolyte, which then forms H_2_O, the intermediate O, and electrons. Then, the intermediate O bonds with the OH^−^ from the solution, and this forms the intermediate OOH and electrons. Finally, the intermediate OOH combines with the OH^−^ and forms H_2_O, O_2_, and electrons [35]. For Ni-based electrocatalyst processes, some studies considered that the optimal bonding of OH and Ni ions formed NiOOH, which is important to the reactive activity in an OER [36].

The addition of Fe was found to introduce a heterostructure to the crystal lattice, which contributed to the electron transport of the electrocatalyst [37,38]. The doping of Mo could introduce higher valence state atoms such as Mo^6+^ and Mo^4+^ to the lattice of the electrocatalyst, which would decrease the bond distance of Ni-M and Ni-O (M represents other metal atom). This contributed to improve the surface area for the catalytic reaction, which benefits to the efficiency of the charge transport in the hydrolysis [39].

**Figure 7:**
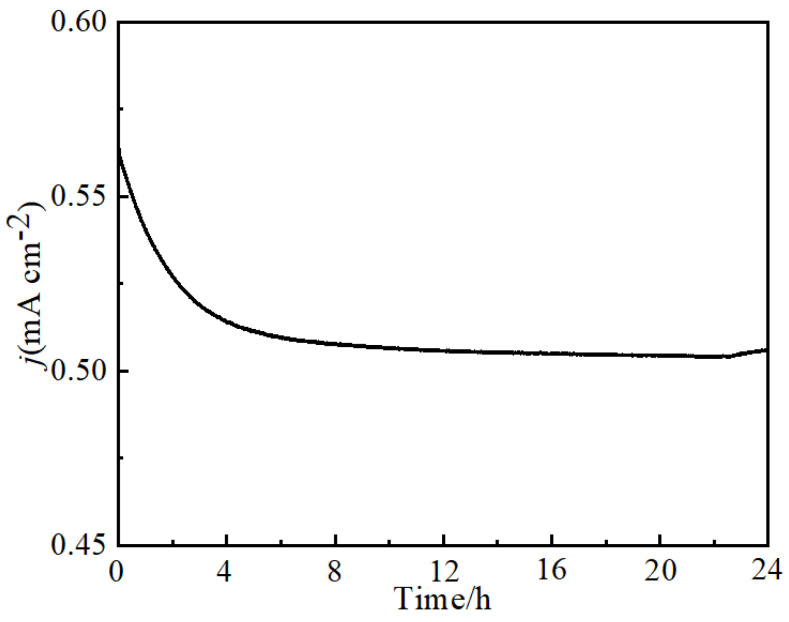
Stability test of the NiFeMo_0.1_-LDH nanosheet arrays.

**Figure 8:**
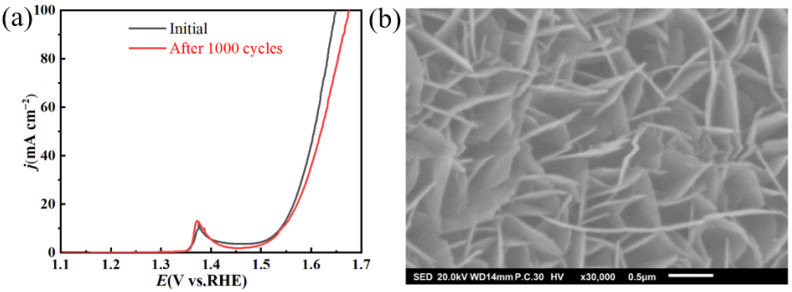
(**a**) LSV curves of NiFeMo_0.1_-LDH in stability test. (**b**) Morphology of NiFeMo_0.1_-LDH after OER.

Stability is crucial to the use of electrocatalysts [40]. The long-term stability of NiFeMo_0.1_-LDH used in the OER was tested using a chronoamperometry method at 1.54 V (vs RHE) for 24h in 1.0 M KOH. The test result in Figure 7 shows that a steady curve of current density–time was obtained. After 1000 CV cycles, there was only a little change that happened to the LSV of NiFeMo_0.1_-LDH when compared to that before the cycle test (Figure 8a). The morphology of the NiFeMo_0.1_-LDH that after the OER proves the crystallinity and microstructure of the NiFeMo_0.1_-LDH remained unchanged after the test (Figure 8b). These indicates the outstanding stability of the NiFeMo_0.1_-LDH.

The comparison of this study with other similar work is shown in Table 3. The special nanoarray morphology of the NiFeMo-based LDH provided a substantial active surface for the OER, which contributed to the more competitive overpotential at 10 mA cm^−2^ and Tafel slopes than most of the reported works. The NiFeMo_0.1_-LDH is a prospective choice for the electrolysis of water.

## 3. Materials and Methods

### 3.1. Materials

Ni foam (NF, purity 99.5%) and Ni(NO_3_)_2_·6H_2_O (99.8%), Fe(NO_3_)_3_·9H_2_O (99.5%), Na_2_MoO_4_·2H_2_O (99.8%) were supplied by Aladdin (China). Ethyl alcohol, CO(NH_2_)_2_, KOH, and HCl were purchased from Sinopharm Group.

### 3.2. Preparation of Ni-Fe-Mo Nanosheet

Ni foams (NF, 2cm × 2cm) were ultrasonically cleaned for 30 min in HCl solution. After being washed by distilled water for several times, the NF was vacuum-dried for 10 h. The next step was preparing Ni-Fe-Mo-based LDH. Ni(NO_3_)_2_·6H_2_O, Na_2_MoO_4_·2H_2_O, Fe(NO_3_)_3_·9H_2_O, and CO(NH_2_)_2_ were dissolved in 100 mL distilled water and stirred for 30 min. The ratios of Ni: Fe: Mo (molar ratio) were kept to 1:1:0, 1:1:0.1, 1:1:0.2, and 1:1:0.3. CO(NH_2_)_2_ was 5 mmol in quantity. Then, the above solutions and the foams were transferred to polytetrafluoroethylene reactors with steel shell. The reactors were kept for 5 h at 150 °C. When the reactors cooled down, the samples were washed by distilled water and ethanol several times. After that, the samples were vacuum-dried for 10 h at 80 °C.

The electrochemical performance of the NiFeMo based-LDH could be optimized by adjusting the atomic ratio of Ni: Fe: Mo. The specimens were marked as NiFe-LDH, NiFeMo_0.1_-LDH, NiFeMo_0.2_-LDH, and NiFeMo_0.3_-LDH.

### 3.3. Structural Characterization

X-ray diffraction (XRD, D8 Advance, Bruker) with Cu Kα radiation was used to detect the phase of the electrocatalysts, the scanning speed was 5/min, the 2θ range was 10–80 degree, and the wavelength was 0.154056 nm. After the hydrothermal reaction, the NFs were sonicated in distilled water for several hours in an ultrasonic washer. Then, the exfoliated powders were collected and vacuum-dried, and they were used to the XRD test. Morphologies and EDS were investigated using field emission scanning electron microscope (JSM7800F, JEOL Ltd., Tokyo, Japan) and transmission electron microscopy (JEM2100, JEOL Ltd., Tokyo, Japan), and the accelerating voltages were 20 kV and 200 kV, respectively. The pore volume and specific area of the electrocatalysts were examined by a specific surface area and porosity analyzer (JW-BK222, JWGB Ltd., Beijing, China). The chemical valent states of the electrocatalysts were analyzed by an X-ray photoelectron spectroscopy (Thermo Fisher Scientific, Shanghai, China).

### 3.4. Electrochemical Performance

The electrochemical tests were carried out in a three-electrode system with a Tesco CHI 660D (Tesco, Shanghai, China) electrochemical workstation. The as-obtained electrocatalysts, a platinum electrode and an Ag/AgCl electrode, and were used as the working electrodes, being the counter electrode and the reference electrode, respectively. The electrolyte was 1M KOH solution. The working electrode potentials were converted to reversible hydrogen electrodes (RHEs) according to the following formula: E_RHE_ = E_Ag/AgCl_ + 0.1989 + 0.0591 × pH. The overpotentials could be calculated according to formula: η(V) = E_RHE_ − 1.23. Linear sweep voltammetry (LSV) measures of the material were tested at 2 mVs^−1^ and with iR compensation. Cycle voltammetry (CV) measures were measured at the range 1.0–5.0 mV/s. Electrochemical impedance spectroscopy (EIS) was recorded from 0.01–10^4^ Hz. Stability test was carried out by chronoamperometry at 10 mA cm^−2^.

## 4. Conclusions

In conclusion, we have developed a novel NiFeMo based-LDH electrocatalyst using a cost-effective method. The unique nano-microstructure endows the electrocatalyst with more active sites for an OER. The NiFeMo_0.1_-LDH exhibited enhanced activities towards OERs compared to NiFe based-LDHs, and it only required an overpotential of 227 mV for 10 mA cm^−2^ and a Tafel slope of 54.8 mV/dec in 1 M KOH. The excellent performance of the NiFeMo_0.1_-LDH can be ascribed to its unique nanosheet arrays, as well as the synergistic effect of the Ni-Fe-Mo system. These contribute to its large active area, efficient electron transport, and improved reaction kinetics in an OER. This work is expected to provide a new route to design and fabricate low-cost Ni-based electrocatalysts for water electrolysis.

## Figures and Tables

**Table 1:** Average parameter of NiFeMo-based LDHs.

Specimen	Length (µm)	Thickness (µm)	L/T
NiFe-LDH	2.37	0.20	11.8
NiFeMo_0.1_-LDH	1.91	0.13	14.7
NiFeMo_0.2_-LDH	1.53	0.11	13.9
NiFeMo_0.3_-LDH	1.22	0.09	13.5

**Table 2:** Pore volume and specific surface area of NiFeMo_0.1_-LDH.

Sample	Pore Volume (cm^3^/g)	Specific Surface Area (m^2^/g)
NiFeMo_0.1_–LDH	0.003	7.970

**Table 3:** Comparison of this study with other similar works.

Electrocatalyst	Current Density (mA cm^−2^)	Overpotential (mV)	Tafel Slopes (mV/dec)	Reference
NiFe-LDH	10	259	45.8	[41]
Ni(OH)_2_	10	595	87	[42]
RuO_2_/NiFe-LDH/NF	10	226	77.4	[43]
NiCo hydroxide	10	460	65	[44]
CeO_2_-Ni_3_S_2_/NF	10	251	60	[45]
Fe-NiS_2_@NC	10	255	77	[46]
NiCo-NS	10	334	41	[47]
NiFeCo-LDH porous	10	290	152	[48]
NiFeW-LDH	10	270	46	[49]
NiFeMo-LDH	10	227	54.8	This work

## Data Availability

The data presented in this study are available in the article.
